# Low‐Cost, Unsinkable, and Highly Efficient Solar Evaporators Based on Coating MWCNTs on Nonwovens with Unidirectional Water‐Transfer

**DOI:** 10.1002/advs.202101727

**Published:** 2021-08-11

**Authors:** Yaqin Zhu, Guangliang Tian, Yiwen Liu, Haoxuan Li, Pengcheng Zhang, Lei Zhan, Rui Gao, Chen Huang

**Affiliations:** ^1^ Engineering Research Center of Technical Textiles Ministry of Education College of Textiles Donghua University Shanghai 201620 China; ^2^ Key Laboratory of Eco‐Textiles (Ministry of Education) Nonwoven Technology Laboratory Jiangnan University Wuxi 214122 China; ^3^ Shanghai Investigation Design and Research Institute Co. Ltd. Shanghai 200434 China; ^4^ Changzheng Hospital Second Affiliated Hospital of Second Military Medical University Shanghai 200003 China

**Keywords:** interfacial water evaporation, nonwovens, poly(propylene)/polyethylene fibers, solar energy, unidirectional water‐transfer

## Abstract

Solar vapor generation technology is promising in seawater desalination, sewage purification, and other fields. However, wide application of this technology is still largely confined due to its high cost and difficulties for scalable production. In this study, an ever‐floating solar evaporator is fabricated by coating multiwall carbon nanotubes on a bicomponent nonwoven composed of polypropylene/polyethylene core–sheath fibers. This all‐fiber structure is highly porous and ultralight, with large specific area (for efficient water evaporation), interconnected channels (for easy vapor escape), and low thermal conductivity (to avoid heat loss). The unique unidirectional water‐transfer behavior of the nonwoven enables it to spontaneously pump an adjustable amount of water for interfacial solar heating and a delicate balance between water supply and loss may accelerate the evaporation speed of water. These distinct benefits endow the solar evaporator with excellent evaporation rates of 1.44 kg m^–2^ h^–1^ under the simulated irradiation of 1 sun and 12.81 kg m^–2^ d^–1^ under natural sunlight. Moreover, the evaporator can be fabricated by using low‐cost materials and industrialized methods (overall cost ≈2.4 USD m^−2^), making one believe its practical significance for commercial solar steam evaporation.

## Graphical abstract

An ever‐floating and durable solar evaporator is fabricated by coating multiwall carbon nanotubes on an unidirectional water‐transfer nonwoven composed of low‐cost poly(propylene)/polyethylene core–sheath fibers. The evaporator is capable of self‐pumping water. The balance between water supply and evaporation and the using of industrialized method help promote the applization of interfacial solar water evaporation.

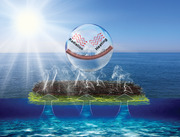

## 1 Introduction

Solar energy is the most promising source of renewable energy on the earth, which is nearly inexhaustible and can be converted into light, heat, or electricity through various methods.^[^ ^1^ ^]^ Among these, utilizing solar energy for water evaporation has been attracting considerable research attention due to its unique advantages such as sustainability, easy‐to‐operate, and free fossil fuel consumption.^[^ ^2^ ^]^ Compared with conventional solar driven evaporators that usually heat volumetric water and suffer from complex and expensive operative conditions,^[^ ^3^ ^]^ solar driven interfacial steam generation have been proposed to reduce energy loss and heat localization at the air–water interface. Recently, pursuing a higher evaporation rate (ER) has impelled the rapid development of solar steam generation with smart structures. A number of inorganic and organic absorbers, such as semiconductor nanomaterials,^[^ ^4^ ^]^ plasmonic nanoparticles,^[^ ^5^ ^]^ and carbon‐based materials and molecules,^[^ ^6^ ^]^ have been proposed as media to convert sunlight into heat and most of these absorbers were combined with floating structures for achieving highly effective interfacial evaporation. Representative works include depositing a thin layer of Al on nanoporous membrane,^[^ ^7^ ^]^ incorporating carbon nanotubes in a polyacrylonitrile nonwoven,^[^ ^8^ ^]^ carbonized mushroom with an umbrella‐shaped black pileus,^[^ ^9^ ^]^ hierarchically nanostructured gel,^[^ ^10^ ^]^ and 3D all‐fiber aerogels.^[^ ^11^ ^]^ All of these designs showed superior capacity of solar steam generation.

Despite the high evaporation efficiencies these studies have achieved, till now, only few solar steam generation systems were designed from the commercial perspective and the development of low‐cost and large‐scale strategies for promoting solar driven interfacial evaporators into industrialization was scarcely explored. Specifically, an interfacial solar water evaporator for commercial purpose is expected to meet a few basic demands, including low‐cost, easy to scale up, long‐term stability and durability, as well as desired evaporation performances. These features are especially important for sea water desalination, which is usually achieved by large numbers of evaporators to float on the sea for harvesting sufficient clean water.^[^ ^12^ ^]^ Therefore, there is a major tendency in using carbon‐based materials with *π* − *π* energy level structure and natural black in solar steam generation system,^[^ ^13^ ^]^ which showed broadband light absorption, easy fabrication, and stable chemical properties. For instance, Li's group^[^ ^14^ ^]^ sprayed graphite solution on a preheated nonwoven mat, which was then wrapped on a polystyrene foam to form an interfacial solar vapor generation device. Owning to the low thermal conductivity (TC) of foam, the evaporation area was separated from bulk water so that less thermal energy was dissipated to the water. To this circumstance, sustaining an adequate water supply from bulk water to evaporation area became another challenge. Fully hydrophilic structure^[^ ^15^ ^]^ provided good capillary force to continuously transport water, but a large water content gathered on the evaporation surface increased the heat consumption speed. Meanwhile, fully hydrophobic structure^[^ ^16^ ^]^ without the formation of “small puddle” faced the problem of large surface tension and thereby imperfect transmission of moisture. To this end, we report for the first time the large‐scale and low‐cost fabrication of an unsinkable solar evaporator, which possesses the ability of self‐pumping water to balance water content on the evaporation surface. Differing from carbon foam,^[^ ^17^ ^]^ hydrogel,^[^ ^18^ ^]^ wood,^[^ ^19^ ^]^ and other floating structures, the multiwall carbon nanotubes (MWCNTs) coated on a nonwoven made of polypropylene/polyethylene (PP/PE) bicomponent fibers further resulted in a rougher, larger, and darker surface to absorb sunlight. When used to evaporate water, the large specific surface area and the interconnected pores of this all‐fiber structure provided excellent thermal management and continuous steam escaping, making it promising for practical application.

## 2 Results and Discussion

### 2.1 Preparation and Characterizations of MWCNT‐Nonwovens

**Figure 1:**
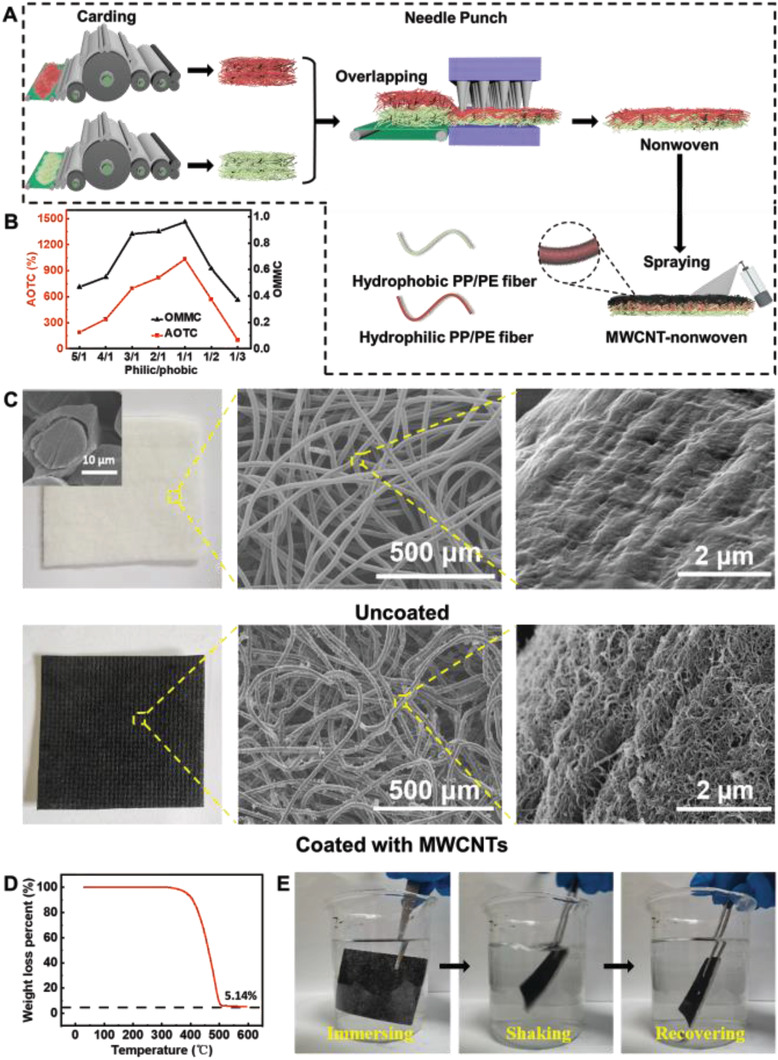
Preparation and characterization of MWCNT‐nonwovens. A) Schematic illustration of the fabrication route of MWCNT‐nonwoven with unidirectional water‐transfer feature. B) AOTC and OMMC of nonwovens composed of different ratios of hydrophilic/hydrophobic fibers. C) Photographs and SEM images of nonwoven (inset is the cross‐section SEM image of PP/PE fiber) with and without MWCNTs. D) Thermogravimetric analysis of MWCNT‐nonwoven and E) stability of the MWCNT‐nonwoven in the simulated sea water.

In a typical experiment (**Figure** **1A**), hydrophilic PP/PE fibers and hydrophobic PP/PE fibers were separately processed into webs by using a pilot carding equipment. A hydrophilic web and a hydrophobic web were then overlapped with each other and consolidated into a nonwoven composite through needle punch technology. Needle punch is a widely adopted nonwoven bonding process, during which the felting needles vertically punch the fibers to make them entangle together.^[^ ^20^ ^]^ When the needles started to penetrate from the hydrophilic side, a proportion of hydrophilic fibers were carried downward to entangle with the hydrophobic fibers from the lower layer. As the needles retreated from the fiber layer, these hydrophilic fibers stayed at the lower layer. After punching twice from each side, the cross‐section of the nonwoven composite changed from two distinct layers to a transitional structure, in which a small proportion of hydrophilic fibers were punched into the hydrophobic majorities, and vice versa. Therefore, a gradient distribution of hydrophilic/phobic fibers was obtained along the thickness direction. This structure is similar to the one in our previous report,^[^ ^21^ ^]^ indicating the existence of a wettability gradient across nonwoven thickness. Considering cost, processability, and requirements from solar water evaporation, the areal density of nonwoven was set at 120 g m^−2^.

Recent progress in water transmission of different dimensions in textiles offered new strategies to develop floating structure for water evaporation such as hanging woven fabric,^[^ ^22^ ^]^ electrospun mat,^[^ ^23^, ^24^ ^]^ and Janus membranes.^[^ ^25^ ^]^ By looking through the advantages and limitations of these materials, we hypothesize that solar steam generation can be effectively accelerated if the evaporator can self‐pump the water with a pumping speed exactly matches the ER of water. Therefore, we adjusted the weight ratio between hydrophilic and hydrophobic fiber webs (**Table** **1**) and compared the unidirectional water‐transfer speed of resultant nonwovens by moisture management tests (MMT). In MMT, accumulative one‐way transport capacity (AOTC) refers to the difference of water content at top surface (hydrophobic side) and bottom surface (hydrophilic side) of materials, and overall moisture management capacity (OMMC) represents the comprehensive performances (top absorption rate, AOTC and wetting time of top surface) of the dynamic transfer of water. According to Figure 1B and Figure S1 in the Supporting Information, both AOTC and OMMC curves show gradual increments when the ratio of hydrophilic/hydrophobic fibers decreased from 5/1 to 1/1, whereas further increasing the content of hydrophobic fibers resulted in the decrease of AOTC and OMMC. As listed in Table S1 in the Supporting Information, desired unidirectional water‐transfer capacity would be achieved when AOTC and OMMC are larger than 400 and 0.8, respectively. Thus, samples with hydrophilic/hydrophobic ratio from 3/1 to 1/1 were selected for the following experiments. For all these samples, water exerted from the hydrophobic side successfully penetrated to the hydrophilic side, but was held back from the opposite direction (Figure S2, Supporting Information). Water penetration time of these samples varied with the ratio of hydrophilic/hydrophobic fibers (Figure S3, Supporting Information).

**Table 1:** Weight ratios of hydrophilic and hydrophobic PP/PE fiber webs

Sample code	Content of hydrophilic fibers [g m^–2^]	Content of hydrophobic fibers [g m^–2^]	Weight ratio
Philic/phobic‐5/1	100	20	5/1
Philic/phobic‐4/1	96	24	4/1
Philic/phobic‐3/1	90	30	3/1
Philic/phobic‐2/1	80	40	2/1
Philic/phobic‐1/1	60	60	1/1
Philic/phobic‐1/2	40	80	1/2
Philic/phobic‐1/3	30	90	1/3
Philic/phobic‐1/4	24	96	1/4
Philic/phobic ‐1/5	20	100	1/5

For the design of a commercial solar evaporator, cost, durability, stability, and evaporation efficiency should be comprehensively considered. We selected MWCNTs as photothermal material due to its broad light absorption wavelength, easy affordability, and superior photothermal conversion capacity (Figure S4, Supporting Information). The MWCNTs were additionally modified with carboxyl, which enables them to better disperse in ethanol solvent and form more hydrogen bonds to strengthen the attachment between MWCNTs and fibers. Photographs and scanning electron microscopy (SEM) images in Figure 1C demonstrate the uniform adherence of MWCNTs on fiber surface and the porous all‐fiber structure, which is critical for water transportation and vapor escape. Raman spectra (Figure S5, Supporting Information) further confirm the successful coating of MWCNTs on the nonwoven, in that two characteristic peaks at 1360 and 1560 cm^−1^ (which are attributed to the D and G bands of carbon, respectively^[^ ^26^ ^]^) are clearly observed. We also found that a total proportion of 5.14% MWCNTs (Figure 1D) was attached on the surface of PP/PE fiber (wherein PE forms the sheath layer and PP forms the core layer, see the inset of Figure 1C) and no loss of MWCNTs was observed when immersing and shaking the nonwoven in simulated sea water (Figure 1E). The reason for such tight attachments lies in the distinct core–sheath structure of PP/PE fiber. When heated with a temperature (120 ℃) slightly higher than the melting point of PE (110 ℃), fiber sheath was melted and served as glue to stick neighboring MWCNTs, thus leading to the firm anchoring of MWCNTs of fiber surface (Figure S6, Supporting Information).

**Figure 2:**
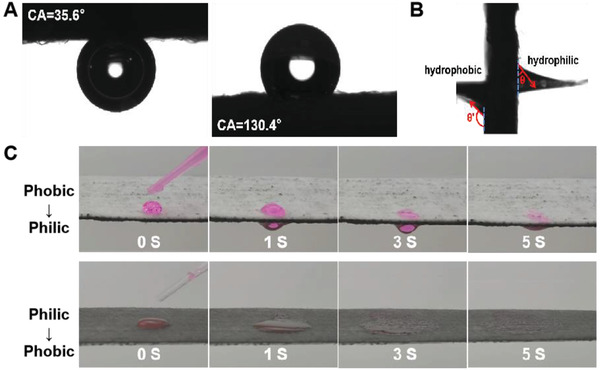
Unidirectional water‐transfer behavior of MWCNT‐nonwoven. A) Representative CA of the hydrophilic and hydrophobic surfaces of MWCNT‐nonwoven. B) Representative cross‐sectional view of the capillary rise on a vertically placed MWCNT‐nonwoven in water. C) Unidirectional water‐transfer of MWCNT‐nonwoven composed of 50% hydrophobic fibers and 50% hydrophilic fibers.

The MWCNT‐nonwoven shows a typical Janus structure with the contact angels (CA) of 130.4° at hydrophobic side and 35.6° at hydrophilic side (**Figure** **2A**). Due to the gradient distribution of hydrophobic and hydrophilic fibers across nonwoven thickness, wettability gradually strengthened from hydrophobic side to hydrophilic side, generating a differential capillary effect (difference in *θ* angle, Figure 2B) to induce the unidirectional water‐transfer.^[^ ^27^ ^]^ Figure 2C shows the unidirectional water‐transfer behavior of the MWCNT‐nonwoven composed of 50% hydrophobic fibers and 50% hydrophilic fibers. When water droplet was exerted from the hydrophobic side, it could spontaneously penetrate through the nonwoven within 5 s. On the contrary, when dropping water from hydrophilic surface, the vertical penetration of water was replaced by the horizontal spread on hydrophilic surface, proving the distinct unidirectional water‐transfer ability of the MWCNT‐nonwoven.

### 2.2 Tunable Water Evaporation Performances of MWCNT‐Nonwoven

**Figure 3:**
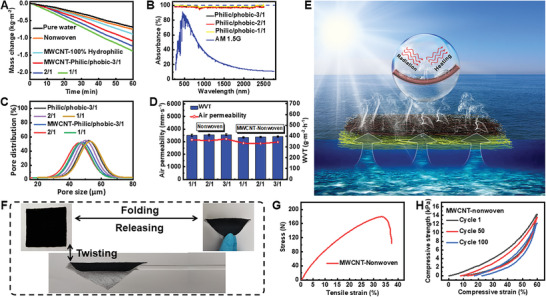
The influence of different weight ratios of hydrophilic and hydrophobic fibers on the properties of MWCNT‐nonwoven. A) Mass loss of water over time under 1 simulated sun. B) The UV–vis–NIR absorption spectrum of MWCNT‐nonwovens (AM1.5G represents the standard solar spectrum). C) Pore size and D) air permeability and WVT of nonwovens and MWCNT‐nonwovens with different ratios of hydrophilic and hydrophobic fibers. E) Schematic diagram of solar steam generation of MWCNT‐nonwoven. F) Bending performance, G) tensile stress, and H) compressive strength of MWCNT‐nonwoven composed of 60 g hydrophilic fibers and 60 g hydrophobic fibers. Error bars indicate SD. ***, *P* < 0.001 (*n* = 5, one‐way ANOVA and Tukey posttest).

To find the optimal ratio of hydrophilic and hydrophobic fibers, water evaporation properties of MWCNT‐nonwovens were measured by placing the samples on bulk water under the irradiation of 1 sun. The hydrophilic side (which was coated with MWCNTs) was exposed to sun for absorbing and converting sunlight into heat. As can be seen in **Figure** **3A**, compared to the control groups (pure water, hydrophilic MWCNT‐nonwoven, and unidirectional water‐transfer nonwoven without MWCNTs), MWCNT‐nonwovens having unidirectional water‐transfer features exhibit faster evaporation, owning to the coating of MWCNTs in combination with the interconnected porous structure. When gradually decreasing the weight ratio of hydrophilic/hydrophobic fibers from 3/1 to 2/1 and 1/1, the corresponding mass change of water increased from 1.09 to 1.24 and 1.44 kg m^−2^ in 1 h, implying that the MWCNT‐nonwoven containing 50% hydrophilic fibers and 50% hydrophobic fibers is the most efficient for solar steam generation. In a standard solar spectrum of Air Mass (AM) 1.5 G (Figure 3B), all the MWCNT‐nonwovens possess similar broadband light absorption of ≈97.6%, with an ultralow transmittance and reflectance of ≈1.3% and ≈1.1% at the wavelength range of 250–2500 nm (Figures S7 and S8, Supporting Information). The pore size and channeling structures of nonwoven before and after coating with MWCNTs are also barely changed (Figure 3C), leading to almost identical air permeability and water vapor transmission (WVT, Figure 3D). These results clearly suggest that except for the ratio of hydrophilic/hydrophobic fibers, the general properties of MWCNT‐nonwovens, including light absorption, pore size, air permeability, and WVT are highly comparable. Given that the change of TC is also negligible before and after coating (Figure S9, Supporting Information), it is rational to ascribe the difference in mass change of water to the change of unidirectional water‐transfer speed. As illustrated in Figure 3E, under sunlight irradiation, water evaporates from the top side of nonwoven. Since the intensity of sunlight and the photothermal conversion capacity of MWCNTs are constant, the key for achieving fast evaporation is to confine the heat generated by MWCNTs on the top surface of nonwoven and utilize it to evaporate the water in this restrained area. The unidirectional water‐transfer behavior of our nonwoven ensures a continuous pumping of water for evaporation and simultaneously suppresses the heat dissipation to bulk water. More importantly, once the amount of water pumped by nonwoven (which can be readily adjusted by regulating the ratio of hydrophilic and hydrophobic fibers) matches the amount of evaporated water, a delicate synergy between water supply and water loss could be reached, leading to effective utilization of heat and thus efficient evaporation of water. For the MWCNT‐nonwoven containing 60 g hydrophilic fibers and 60 g hydrophobic fibers, MMT analyses (Figure S1, Supporting Information) and water self‐pumping photograph (Figure S10, Supporting Information) suggest that the unidirectional water‐transfer speed is 23.4 mL m^−2^ min^−1^. This value is very close to the evaporation results (24.0 g m^−2^ min^−1^), implying that the balance between water supply and evaporation can effectively accelerate the ER.

The MWCNT‐nonwoven with highest ER was further selected for stability tests. As presented in Figure 3F (see also Video S1 and S2, Supporting Information), it could return to original shape after repeated folding and twisting, with a high specific stress of ≈176 N (Figure 3G). The cyclic compression tests show that the deformation of MWCNT‐nonwoven was 10.15% at 1st cycle, and 22.43% at 100th cycles, respectively (Figure 3H). No significant variation in stress and compression were observed before and after coating of MWCNTs (Figure S11, Supporting Information). These outstanding mechanical properties offer long‐term working stability and may help the solar evaporator to withstand harsh environments (such as storms and large waves) when used in applications such as sea water desalination.

**Figure 4:**
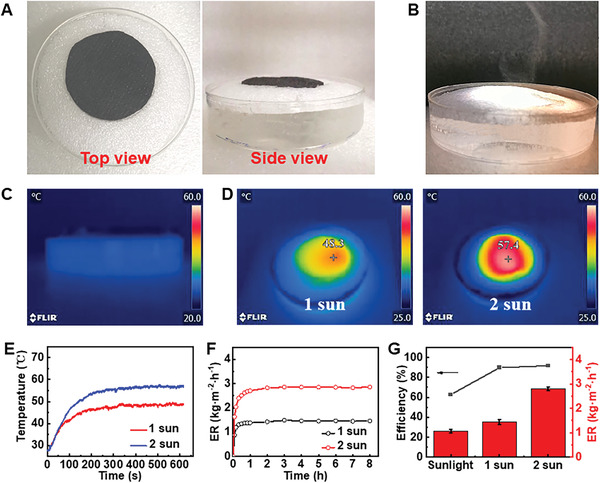
Water evaporation performances of selected MWCNT‐nonwoven under simulated sunlight. A) Photographs of the evaporation system based on MWCNT‐nonwoven. B) Vapor generation of MWCNT‐nonwoven under irradiation of 2 sun. C) Side view IR image of the evaporation system under solar irradiation of 1 sun. D) Top view IR images of MWCNT‐nonwoven under the simulated irradiation of 1 and 2 sun. E) Surface temperature change and F) ER of the evaporation system under the simulated irradiation of 1 and 2 sun over time. G) ER (right‐hand side axis) and corresponding efficiency (left‐hand side axis) of the evaporation system under different irradiation intensities. Error bars indicate SD. ***, *P* < 0.001 (*n* = 5, one‐way ANOVA and Tukey posttest).

Photothermal transfer capacity of the MWCNT‐nonwoven in dry state was studied under the irradiation of various intensity. The surface temperature increased to 76 and 130 ℃ in 100 s irradiation of 1 and 2 sun, respectively (Figure S12, Supporting Information). This indicates that our sample may possess excellent capacity to generate solar steam. As a proof of concept, the MWCNT‐nonwoven was placed in a petri dish filled with 50 mL of simulated sea water, and the edge of the sample was wrapped with foam to avoid extra heating of surrounding water (**Figure** **4A**). It can be clearly seen from Figure 4B that large amount of vapor was generated under simulated sun. The corresponding temperature and the mass loss of water were measured by an IR camera and a real time balance. As shown in Figure 4C, the heat transfer from MWCNT‐nonwoven to bulk water could be blocked, since no obvious temperature change was detected from the side view of evaporator. Notably, heat generated by MWCNTs was highly localized on the surface of evaporator, making the surface temperature of evaporator increase swiftly and became stable at 48 and 57 ℃ under the irradiation of 1 and 2 sun, respectively (Figure 4D,E). In comparison, the temperature of bulk water remained at 25 ℃ throughout the experiment. The elevated temperature at nonwoven surface led to a high and stable ER of 1.44 kg m^−2^ h^−1^ after 2 h of 1 sun irradiation (Figure 4F). To our knowledge, this rate is superior to most of the previous reports^[^ ^28^, ^29^, ^30^ ^]^ and can be further increased to 2.83 kg m^−2^ h^−1^ when enhancing the solar irradiation to 2 sun.

To evaluate the capability of solar vapor evaporation, the conversion efficiency of solar to steam generation is calculated by the following equations^[^ ^5^ ^]^

(1) $$\eta =\frac{mh_{\mathrm{lv}}}{C_{\mathrm{opt}}P_{0}}$$

where *m* denotes the mass flux, *h*_lv_ denotes the total enthalpy of liquid‐vapor change including sensible heat and latent heat, *C*_opt_ denotes the optical concentration, and *P*_0_ is the solar irradiation at the power density of 1 kw m^–2^.

(2) $$m=m_{\mathrm{solar}}-m_{\mathrm{dark}}$$

where *m*_solar_ is the ER under solar irradiation and *m*_dark_ is the ER under dark circumstance.^[^ ^11^ ^]^ As mentioned above, *h*_lv_ contains two part: the sensible heat and the phase change enthalpy (*h*_lv_ = *C* (*T* − *T*_0_) + △*h*_vap_).^[^ ^31^ ^]^ In our study, *m*_solar_ and *m*_dark_ were 1.44 and 0.15 kg m^−2^ h^−1^. After substituting values and converting units, evaporation efficiency of our MWCNT‐nonwoven under 1 sun was 89.7%, and would increase to 91.7% when the irradiation increased to 2 sun (Figure 4G). The excellent conversion once again confirms synergetic benefits of intensive sunlight harvest, efficient thermal management, and most importantly the delicate balance between water supply and water loss of the MWCNT‐nonwoven. If the water pumping speed is higher than the evaporation speed, more water would be pumped to nonwoven surface, causing the accumulation of water and thus an inevitable decrease in water temperature.^[^ ^32^ ^]^ By contrast, if the water pumping speed is lower than the evaporation speed, heat cannot be fully utilized. Therefore, the highest solar to steam conversion efficiency would be obtained when the two speeds are well matched, which in our case can be obtained by using the MWCNT‐nonwoven containing 60 g hydrophilic fibers and 60 g hydrophobic fibers.

### 2.3 Real‐Time Outdoor Evaporation of MWCNT‐Nonwoven

**Figure 5:**
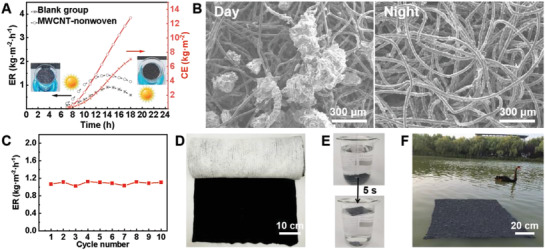
Outdoor evaporation and large‐scale production of MWCNT‐nonwoven. A) ER and cumulative evaporation (CE) of MWCNT‐nonwoven at 7:00 to 18:00 over a day at the average temperature of 27 ℃ outdoors. Insets are the photographs of the MWCNT‐nonwoven at the beginning and the end of the outdoor experiment. B) SEM images of MWCNT‐nonwoven at daytime (with salt particles) and at night (without salt particles), respectively. C) ER of MWCNT‐nonwoven during ten cycles water evaporation under natural sunlight. D) Photograph of a roll of MWCNT‐nonwoven. E) The unsinkable property of MWCNT‐nonwoven: the nonwoven was submerged in water by a glass rod for 30 s and could float to the top within 5 s. F) MWCNT‐nonwoven with the size of 1 m × 1 m floating on a Jingyue lake in Donghua University.

To validate the efficacy of using our MWCNT‐nonwoven as solar evaporator in actual environment, an outdoor experiment was conducted under natural sunlight from 6:00 to 18:00. As shown in **Figure** **5A**, ER was 0.23 kg m^−2^ h^−1^ at 7:00. As time went by, it increased to 0.73 kg m^−2^ h^−1^ at 9:00 and 1.39 kg m^−2^ h^−1^ at 13:00, then decreased to 1.15 kg m^−2^ h^−1^ at 18:00. The average evaporation rate in the whole day was 1.067 kg m^–2^ h^–1^, with a solar to steam generation efficiency (62.7%) higher than most of the commercial solar stills (35–45% in evaporation efficiency).^[^ ^33^ ^]^ We further applied a self‐made water collecting system (Figure S13, Supporting Information) to verify the water collection capacity of the MWCNT‐nonwoven. With an evaporation area of 0.04 m^2^, the collector could harvest ≈0.31 kg of clean water per day. Sea water desalination capacity of the MWCNT‐nonwoven was also investigated by evaporating sea water (East China sea) under natural sunlight. The concentrations of all these ions (Na^+^, K^+^, Mg^2+^, and Ca^2+^) decreased dramatically from 2352, 952, 542, and 689 mg L^−1^ in original seawater to 0.08, 1.22, 1.52, and 0.34 mg L^−1^ in the resultant desalinated water (Figure S14, Supporting Information). The concentration of these ions was much lower than the World Health Organization standard for drinking water (1‰). Despite the inevitable deposition of salt crystals on the surface of nonwoven (Figure 5B), it is able to generate clean water on daytime and self‐clean on night, in that salt crystals can be dissolved by the water pumped at night. As a result of this, no significant change in evaporation performances was found during ten testing cycles using simulated sea water under natural sunlight (Figure 5C).

### 2.4 Large‐Scale Production and Unsinkable Feature of MWCNT‐Nonwoven

When it comes to practical applications, it is essential to scale up the production and reduce the cost. The raw materials of our solar evaporator, i.e., PP/PE bicomponent fibers, have been widely used in hygiene products (such as the superficial layers of diapers and napkins) with up‐to‐date market price of 3.2 USD kg^−1^. Although MWCNTs are relatively expensive (300 USD kg^−1^), they only account for 5.14% in mass of the evaporator (Figure 1D). Moreover, the methods involved in the fabrication of MWCNT‐nonwoven, including carding, needle punching, and solution coating (with pure ethanol as solvent) have all been fully industrialized and the overall processing cost can be easily controlled in less than 0.2 USD m^−2^. Therefore, the total cost of MWCNT‐nonwoven is ≈2.4 USD m^−2^, which can be afforded by most applications. We also conducted the performance comparison of state‐of‐the‐art evaporators (Table S2, Supporting Information). Our sample stands out in the comprehensive aspects of evaporation efficiency, preparation process, and cost. A MWCNT‐nonwoven with the size of 0.5 m × 5 m was fabricated to prove the large‐scale production (Figure 5D). The flexibility was also demonstrated by coiling the MWCNT‐nonwoven into a roll, which also indicates an easy transportation from factory to terminal.

More attractively, the densities of PP (0.91 g cm^−3^) and PE (0.96 g cm^−3^) are both smaller than that of water and the proportion of MWCNTs (2.10 g cm^−3^) was only 5.14%. Based on the density and proportion of these materials, we calculated the actual density of MWCNT‐nonwoven by the equation:

(3) $$\bar{\rho }=\frac{m_{\mathrm{PP}}\rho_{\mathrm{PP}}+m_{\mathrm{PE}}\rho_{\mathrm{PE}}+m_{\mathrm{MWCNTs}}\rho_{\mathrm{MWCNTs}}}{m_{\mathrm{PP}}+m_{\mathrm{PE}}+m_{\mathrm{MWCNTs}}}$$

where $\bar{\rho }$ represents the overall density of MWCNT‐nonwoven and *m* and *ρ* represent the mass and density of the materials, respectively.

Assuming there are no pores in MWCNT‐nonwoven, for 1 m^2^ of the nonwoven, *m*_PP_, *m*_PE_, and *m*_MWCNTs_ are 60, 60, and 6.168 g, respectively. Thus, the overall density of MWCNT‐nonwoven is 0.99 g cm^−3^, which is even lower than the density of water (1.00 g mL^−1^). We believe that this ultralow density is first acquired in fiber‐based solar steam evaporators because most fibers used in previous designs were heavier than water.^[^ ^34^, ^35^, ^36^ ^]^ As a result of this, no matter how long the MWCNT‐nonwoven was immersed in water, it would eventually float on top of the water by itself (Figure 5E, see also Video S3, Supporting Information), indicating an unsinkable feature that is of critical practical significance in natural environments. This is further proved in Figure 5F, in which an MWCNT‐nonwoven with the size of 1 m × 1 m was found to continuously float on a lake.

## 3 Conclusion

In summary, we have demonstrated the low‐cost and scalable fabrication of a solar evaporator based on bicomponent PP/PE nonwoven and MWCNTs. The gradient distribution of hydrophobic and hydrophilic fibers at nonwoven thickness endowed the evaporator with adjustable unidirectional water‐transfer behavior, which was proved critical to reach the balance between water supply and evaporation, and thus greatly promoting the water evaporation speed. The evaporator was unsinkable in water, since the overall density of all components was even lower than that of water. Besides, the sustainable water evaporation in daytime and self‐cleaning at night guaranteed a long service life. We envision that the high flexibility, efficient water evaporation, low‐cost, and mass‐production of MWCNT‐nonwoven may provide new opportunities for pushing solar induced interfacial steam generation into commercial applications.

## 4 Experiment Section

### Materials

PP/PE bicomponent fiber (containing 50% PE fiber as the sheath layer and 50% PP fiber as the core layer) was obtained from Nantong Luolai Chemical Fiber Co., Ltd. Carboxyl functionalized MWCNTs (length ≤ 30 µm, diameter = 15 ± 5 nm) were purchased from Chinese Academy of Sciences. Anhydrous ethanol was provided by Sinopharm.

### Preparation of Nonwoven with Unidirectional Water‐Transfer

The weight ratios of hydrophilic and hydrophobic PP/PE fibers were set from 5/1 to 1/5 (see Table 1). As the area density of nonwoven was set as 120 g m^−2^, the weight of these two types of fibers could be calculated. For example, the philic/phobic‐1/1 refers to the nonwoven composed of 60 g hydrophilic fibers and hydrophobic fibers in 1 m^−2^, respectively. After carding, the hydrophilic fiber layer and the hydrophobic fiber layer were overlapped with each other and were consolidated into a nonwoven composite through needle punching. Each nonwoven was needle punched twice at both sides.

### Fabrication of MWCNT‐Nonwoven

MWCNTs were dispersed in ethanol solution at the concentration of 0.1 wt%, followed by 20 min of ultrasonication. The MWCNTs solution was then sprayed through a commercial atomizer (nozzle diameter = 0.3 mm, Samma, Japan) toward the hydrophilic side of preheated nonwoven. The MWCNT‐nonwoven with a thickness of 3 mm was obtained after drying the sample at 120 ℃ for 10 min.

### Characterization

SEM images of the samples were observed by using SU8010 field emission scanning electron microscope (Hitachi, Japan). Mechanical properties were measured by the Instron 5982 universal material testing system (Instron, USA) and HY‐940FS compressive pressure testing machine (Zhongye Jingke instrument, China). MMT was tested by an M290 moisture management device (SDL Atlas, China). Samples were cut into 8 cm × 8 cm and each test lasted for 2 min. The water used for each test is 0.3 mL. Water contact angel was measured at room temperature by OCA15EC contact angle measuring instrument (Dataphysics, German). Air permeability was measured by YG461E air permeability tester (Ningbo Textile Instrument, China). Moisture permeability was tested by YG601H moisture permeability meter (Ningbo Textile Instrument, China). Pore size was measured by CFP‐1100‐AI Capillary Flow Porometer (Porous Materials Inc., USA). Raman spectra were measured by inVia‐Reflex Raman spectrometer (Renishaw, UK). Thermal conductivity was measured by TC3000E thermal conductivity meter (Jthermo, China). IR thermal images and digital photographs were taken by E6 IR‐camera (Flir, USA) and navo5‐pro mobile phone (Huawei, China), respectively. Absorbance spectra were recorded using PerkinElmer Lambda 950 (PerkinElmer, USA). The concentration of ions was tested by inductively coupled plasma mass spectrometry (Thermo Fisher, USA).

### Solar Steam Generation Measurements

Simulated solar steam generation was performed by a Solar‐500L solar simulator system (NBeT, China), which contains a solar simulator and an AM1.5G light filter. The solar density was calculated by a VLP‐2000 light power meter (Laser, China). The MWCNT‐nonwoven was cut into round shape (*R* = 2.4 cm) and floated on simulated seawater (3.5% of the salt dissolved in deionized water) in a petri dish with the hydrophilic layer exposing to simulated sun. As the water started to be pumped to the top surface of nonwoven, the simulated solar irradiation was turned on and the water mass change was measured by a PR224ZH high accuracy balance (Ohaus, USA). During experiment, the ambient temperature and the relative humidity were 25 ℃ and 60%, respectively. Outdoor solar steam generation was conducted at the roof of College of Textiles, Donghua University. Simulated sea water was added in a petri dish with an MWCNT‐nonwoven (*R* = 2.4 cm) placed on it. The whole evaporation device was put on a real time balance and the data were recorded every hour from 6:00 to 18:00. Further, A self‐made glass cover with inclined walls (length: 30 cm, width: 25 cm, and height: 15–25 cm) was used for water collection (Figure S13, Supporting Information). Sea water (from East China Sea) and a 0.04 m^–2^ of MWCNT‐nonwoven were placed in left chamber. Vapor generated by the evaporator during sunlight was condensed on the top cover and then flowed down to the right chamber.^[^ ^37^ ^]^

### Statistical Analysis

The experiment data of moisture permeability, pore size air permeability, mechanical properties, and evaporation efficiency were presented as the mean ± standard deviation (SD) of 5 (*n*) samples. Data were evaluated by one‐way analysis of variance (ANOVA) followed by a Tukey multiple comparisons posttest. Differences were considered to be statically significant at *p* < 0.001. Data were analyzed by using the Origin and Excel software.

## Conflict of Interest

The authors declare no conflict of interest.

## Supporting information

Supporting Information

Supplemental Video 1

Supplemental Video 2

Supplemental Video 3

## Data Availability

Research data are not shared.
