# Supplementary material for: Low‐Cost, Unsinkable, and Highly Efficient Solar Evaporators Based on Coating MWCNTs on Nonwovens with Unidirectional Water‐Transfer
Source: Adv Sci (Weinh). 2021 Aug 11;8(19):2101727. doi: 10.1002/advs.202101727 (PMC8498870; doi:10.1002/advs.202101727)
Supplement: Supplementary file 1 — Supporting Information [file ADVS-8-2101727-s003.pdf]

WILEY-VCH

© 2021 Wiley-VCH GmbH

ADVANCED SCIENCE
Open Access

Supporting Information

for *Adv. Sci.,* DOI: 10.1002/advs.202101727

Low-cost, Unsinkable and Highly Efficient Solar Evaporators Based on Coating MWCNTs on Nonwovens with Unidirectional Water-transfer

Yaqin Zhu, Guangliang Tian, Yiwen Liu, Haoxuan Li, Pengcheng Zhang, Lei Zhan, Rui Gao,* and Chen Huang**

Y. Q. Zhu, Dr. G. L. Tian, Y. W. Liu, Dr. L. Zhan, Dr. C. Huang
Engineering Research Center of Technical Textiles, Ministry of Education, College of Textiles, Donghua University, Shanghai 201620, China
E-mail: hc@dhu.edu.cn

Prof. H. X. Li
Key Laboratory of Eco-Textiles (Ministry of Education), Nonwoven Technology Laboratory, Jiangnan University, Wuxi 214122, China
E-mail: lihx@mail.dhu.edu.cn

Dr. P. C. Zhang
Shanghai Investigation, Design & Research Institute Co. Ltd., Shanghai 200434, China

Prof. R. Gao

Changzheng Hospital, Second Affiliated Hospital of Second Military Medical University, Shanghai 200003, China
E-mail: gaoruispine@126.com

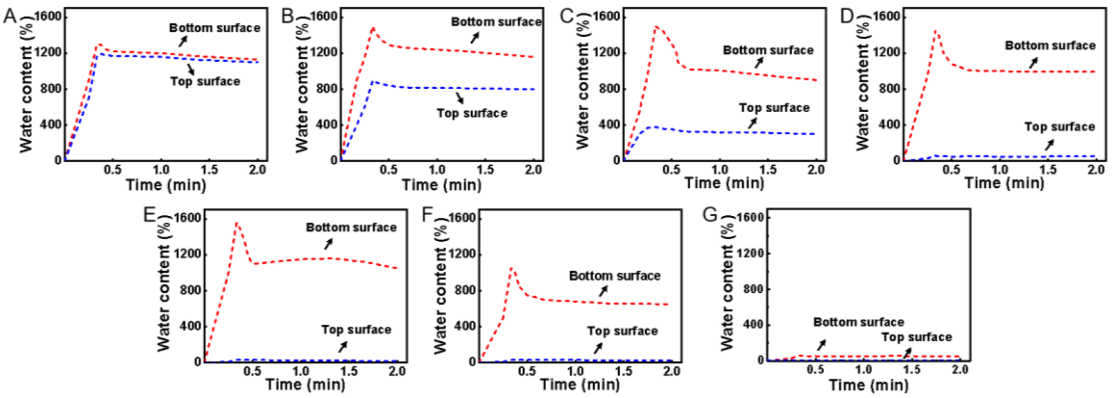

Figure S1. Schematic diagrams of MMT of (A) Philic/phobic-5/1, (B) Philic/phobic -4/1, (C) Philic/phobic-3/1, (D) Philic/phobic-2/1, (E) Philic/phobic-1/1, (F) Philic/phobic-1/2 and (G) Philic/phobic-1/3 nonwovens (The top side is the hydrophobic side).

Without the assistance of external forces, water could not penetrate hydrophobic porous media, since the gravity of water is insufficient to overcome the hydrostatic resistance of the hydrophobic material. According to wettability theory, to achieve water penetration in porous media, the pressure difference at liquid−gas interface should exceed a threshold value. [1-2] Therefore, to activate the spontaneous water-penetration, we consolidated a layer of hydrophilic fibers with a layer of hydrophobic fibers via needle punch. As can be seen in Figure S1 (Supporting Information), the philic/phobic-5/1 sample is entirely hydrophilic, as water contents at the two surfaces are identical. This is because that during needle punch, a proportion of hydrophilic fibers moved vertically to the hydrophobic side, which greatly reduced the actual hydrostatic pressure of hydrophobic layer. Drawn by these hydrophilic fibers, water droplets at both sides exhibited similar horizontal spread and vertical penetration. When the ratio of hydrophobic fibers gradually increased, the nonwoven composites presented typical unidirectional water-transfer behavior. Vertical penetration could still be achieved by the water from hydrophobic side, since water droplets at this side would be drawn by the small proportion of hydrophilic fibers and flow vertically to the other side. However, water from hydrophilic side intended to spread horizontally rather than vertically, because the hydrophilic fibers at this side significantly outnumbered the hydrophobic fibers. Further increase of hydrophobic fibers to philic/phobic-1/3, the nonwoven became fully hydrophobic, indicating that the hydrophobic ratio of our nonwovens should be less than 75%.

The energy transfer behavior of MWCNT-nonwoven include heat conversion, conduction, convection and radiation. Solar energy absorbed by the MWCNT-nonwoven is converted in the form of heat, and is mostly conducted to the water in this small region for generating vapor. The top surface of the MWCNT-nonwoven was then surrounded by heated vapor, making the temperature of

[1] X. Tian, J. Li, X. Wang, *Soft Matter.* **2012**, *8*, 2633−2637.
[2] B. Su, Y. Tian, L. Jiang, *J. Am. Chem. Soc.* **2016**, *138*, 1727−1748.

adjacent environment above the nonwoven close to the temperature of nonwoven surface. Hence，only a small proportion of heat dissipates to ambient environment through the heat convection caused by the flow of vapor steam.

As fiber ratio changes, the MWCNT-nonwovens present different rates of water transfer. When the rate is faster than water evaporation rate, water would accumulate on the surface of nonwovens, and more energy is required to keep the temperature, which inevitably causes energy loss and subsequently a low solar to steam efficiency. In the case that water transfer rate is lower than evaporation rate, the excessive heat would dissipate into the surroundings due to heat convection and radiation. Therefore, a delicate balance of water transfer and water evaporation is beneficial for the utilization of solar energy, whilst in our work, this balance can be achieved through regulating the areal density of nonwoven and the ratio of hydrophilic/phobic fibers.

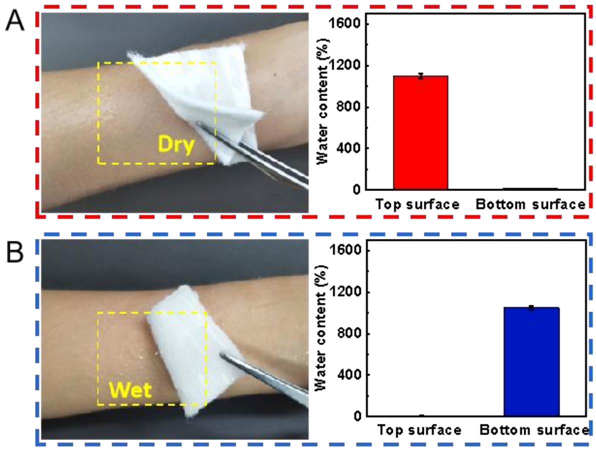

Figure S2. Representative demonstration of nonwovens with unidirectional water-transfer: (A) water could not transfer from hydrophilic side to hydrophobic side. (B) water spontaneously transferred from hydrophobic side to hydrophilic side.

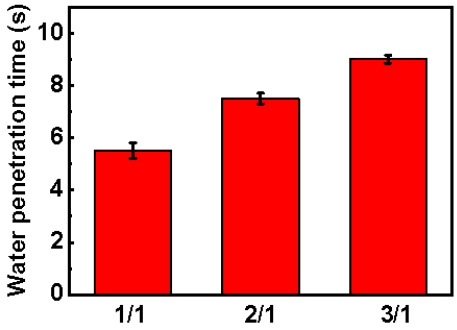

Figure S3. Water penetration time of nonwovens containing different weight ratios of hydrophilic/hydrophobic fibers.

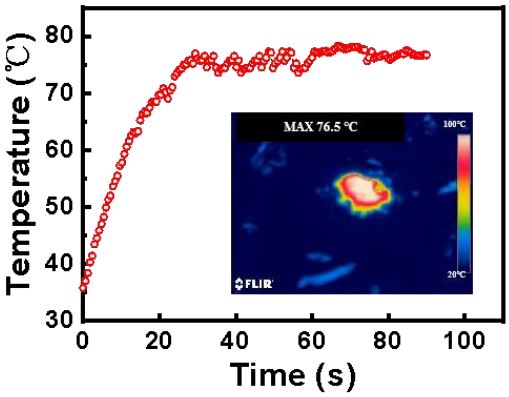

Figure S4. Temperature changes of MWCNTs over time under the irradiation of 1 sun for 100 s. Inset is an IR thermal image of MWCNTs upon exposure to 1 sun for 100 s.

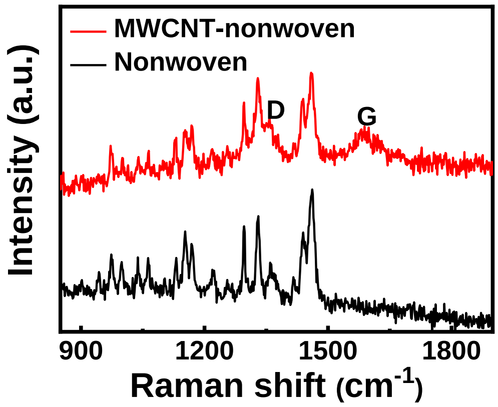

Figure S5. Raman spectra of pure nonwoven and MWCNT-nonwoven.

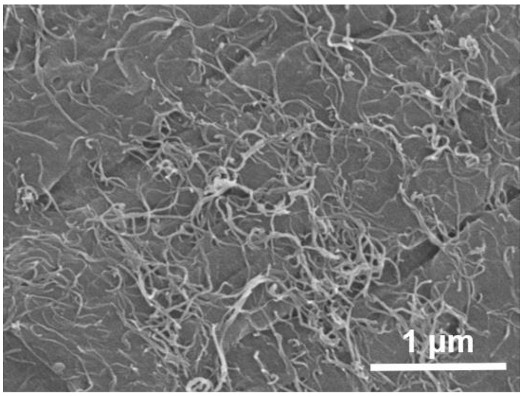

Figure S6. SEM image showing the firm anchoring of MWCNTs of fiber surface.

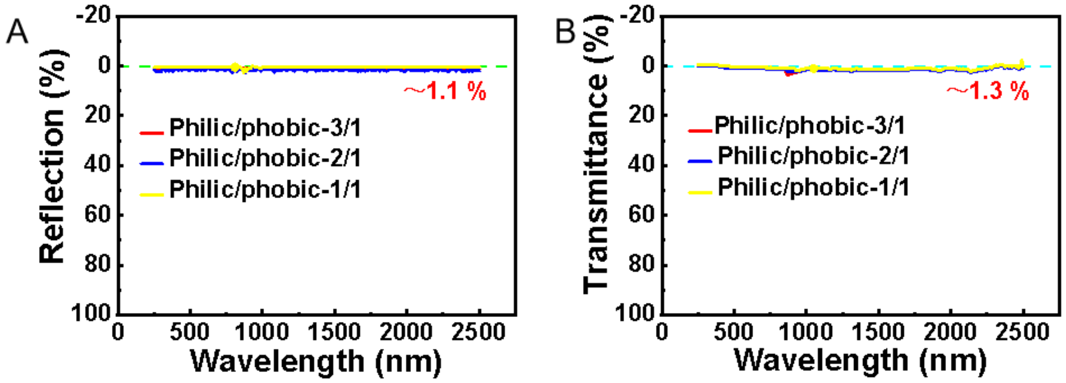

Figure S7. (A) Reflection and (B) transmittance spectrum of MWCNT-nonwovens.

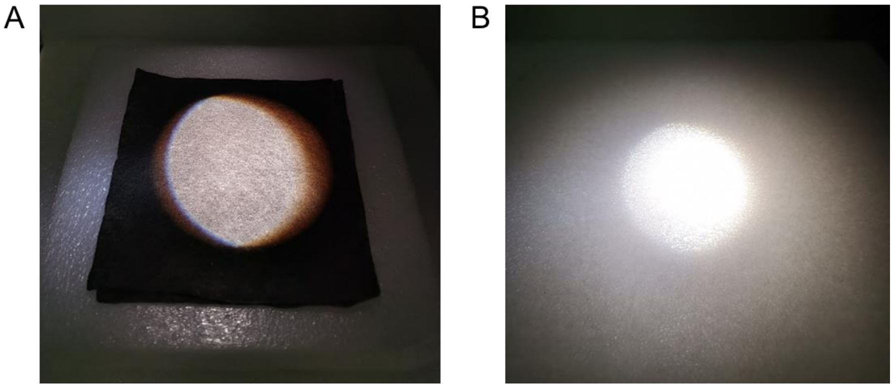

Figure S8. Irradiation image of (A) pure evaporator and (B) foam

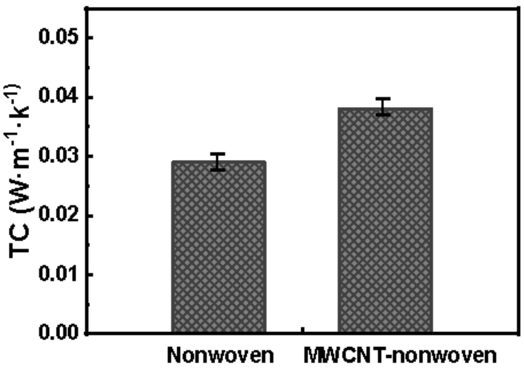

Figure S9. Thermal conductivity (TC) of pure nonwoven and MWCNT-nonwoven.

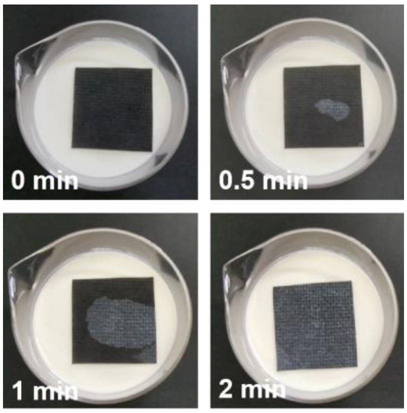

Figure S10. Photos of the MWCNTs-nonwoven containing 60 g hydrophilic fibers and 60 g hydrophobic fibers on the surface of milk (for more obvious contrast due to the dark surface) showing the self-pumping and diffusion of milk to the top surface in 2 min.

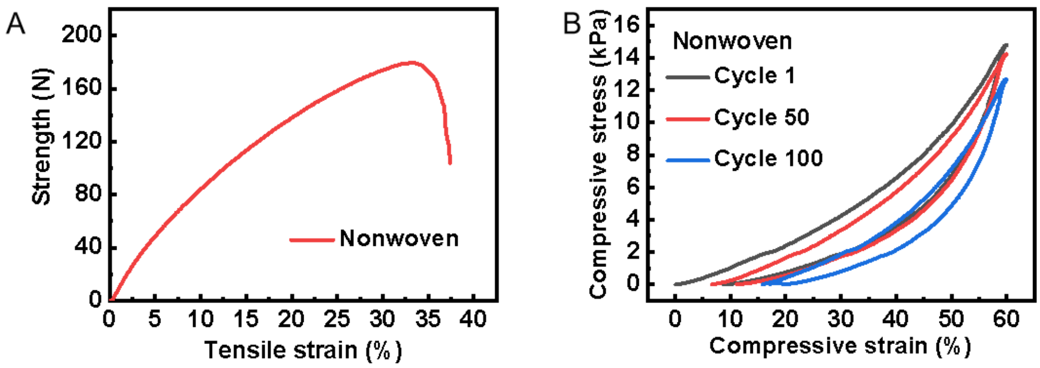

Figure S11. (A) Tensile strength and (B) compressive stress of nonwoven

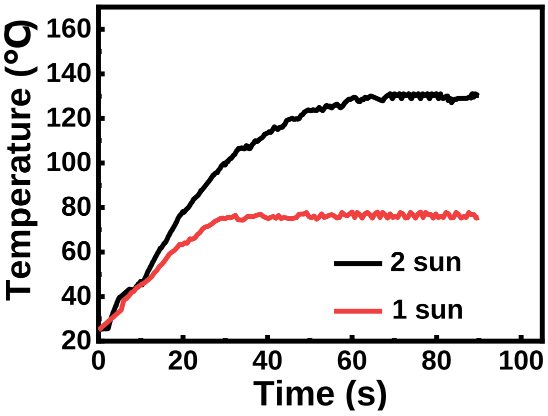

Figure S12. Surface temperature changes of MWCNT-nonwoven in dry state under different solar irradiation intensities.

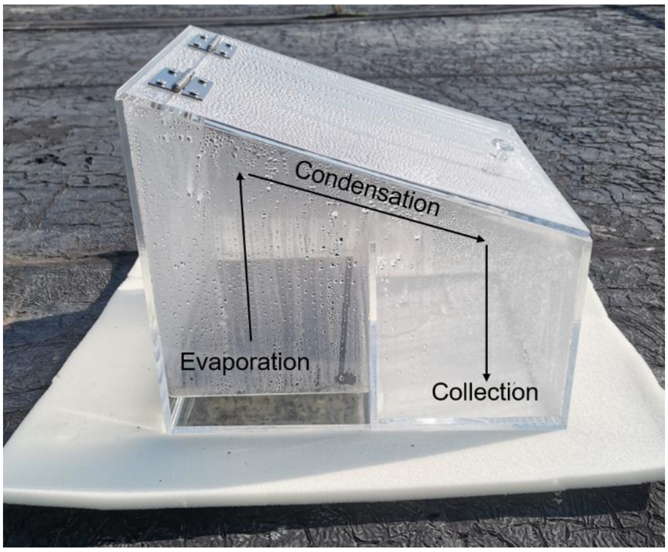

Figure S13. Photograph of the self-developed water collecting system.

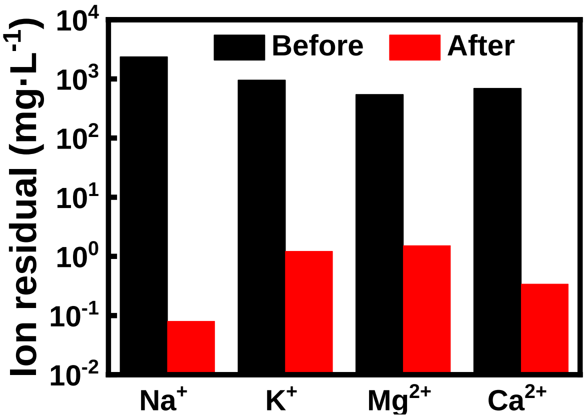

Figure S14. Salinities (the weight percentage of ions) of primary ions Na^+, K^+, $Mg^{2+}$and Ca^{2+} in actual sea water (East China sea) before (original) and after (desalinated) solar thermal desalination.

Table S1. AOTC and OMMC performance rating

Remark	AOTC (%)	OMMC
Terrible	< 50	0 ~ 0.2
Bad	< 50	0.2 ~ 0.4
General	100 ~ 200	0.4 ~ 0.6
Good	200 ~ 400	0.6 ~ 0.8
Excellent	> 400	> 0.8

Table S2. The comparison of different low-cost evaporators

Absorber	Floating material	Evaporation efficiency	Process	cost
Hydrophilic black cellulose fabric[1]	Expanded polystyrene foam	56%	Sewing	\$3 m^{-2}
Self-synthesis AuNP@c-silica[2]	Filter fiber paper	94.6%	Synthesis and coating	> \$18 m^{-2}
Biomass mesoporous carbon[3]	Polystyrene foam	84.95%	Reaction and adhesion	\$39 m^{-2}
Carbon Black Nanoparticle[4]	Air-laid paper and polystyrene foam	53.9%	Vacuum filtration coating	\$16.86 m^{-2}
Al-Ti-O hybrid[5]	PVDF membranes	77.5%	Planetary-milling and coating	\$11.17 m^{-2}
Candle soot[6]	Cotton fabric with polystyrene foam	80%	Dipping coating	< \$10 m^{-2}
Polypyrrole[7]	Fibrous cotton with polystyrene foam	83%	Immersing and polymerization	\$2.75 m^{-2}
Multi-walled carbon nanotubes[8]	Porous cotton fiber paper	43%	Silylation and Coating	\$5.3 m^{-2}
Carbon nanotubes[9]	Cross-linked polycyclic octenes	83%	Crosslinking and etching	\$43 m^{-2}
Our sample	nonwoven	89.7%	Needle punch and coating	\$2.4 m^{-2}

[1] G. Ni, S. H. Zandavi, S. M. Javid, S. V. Boriskina, T. A. Cooper, G. Chen, *Energy Environ. Sci.* **2018**, *11*, 1510.
[2] R. R. Cui, J. l. Wei, C. Du, S. S. Sun, C. Zhou, H. G. Xue, S. Y. Yang, *J. Mater. Chem. A* **2020**, *8*, 13311.
[3] F. H. Liu, B. Y. Zhao, W. P. Wu, H. Y. Yang, Y. S. Ning, Y. J. Lai, R. Bradley, *Adv. Funct. Mater*. **2018**, *28*, 1803266
[4] L. Chen, H. Y Wang, S. Kuravib, K. Kotab, Y. H. Parkb, P. Xua, *Desalination* **2020**, *483*, 114412.
[5] L. C Yia, S. Q. Cia, S. L. Luo, P. Shao, Y. Hou, Z. H. Wen, *Nano Energy* **2017**, *41*, 600–608.
[6] H. M. Wilson, S. Rahman A.R., A. E. Parab, N. Jha, *Desalination* **2019**, *456*, 85–96.
[7] P. Xiao J. C. Gua, C. Zhang, F. Ni, Y. Liang, J. He, L. Zhang, J. Y. Ouyang, S. W. Kuod, T. Chen, *Nano Energy* 2019, 65, 104002.
[8] D. D. Li, Q. X. Zhou, G. Wang, H. Zhao, S. H. Ma, K. Y. Leng, Y. Wang, J. B. Bai, *J. Mater. Sci.* **2020**, *55*, 15551–15561.
[9] L. Y. Zhao, L. Wang, J. D. Shi, X. Y. Hou, Q. Wang, Y. Zhang, Y. Wang, N. N. Bai, J. L. Yang, J. M. Zhang, B. Yu, C. F. Guo, ASC Nano 2021.
